# Russian Innovation in the Era of Patent Globalization

**DOI:** 10.1007/s40319-021-01137-8

**Published:** 2022-01-27

**Authors:** Svitlana Lebedenko

**Affiliations:** grid.15711.33 0000 0001 1960 4179 Ph.D. Researcher, Department of Law, European University Institute, Fiesole, Italy

**Keywords:** Patents, TRIPS Agreement, National innovation systems, Crony capitalism, Russia, Soviet Union

## Abstract

The launch of the Russian Sputnik vaccine in 2020 echoed the launch of the Soviet Sputnik satellite in 1957 and reminded the world once again that Russia is a sophisticated technological power. Most inventions in the Soviet Union were managed by the system of inventor’s certificates which ensured open flows of knowledge among the scientific networks behind Russia’s industrial development. Inventions in today’s Russia are managed by the globalized institution of patents which can create high barriers to entry in innovation markets. This article argues that the globalized institution of patents has been compromised in Russia because the barriers to entry that patents create are not justified in the absence of well-functioning markets. The danger of the institutional mismatch is lost opportunities for Russia to grow knowledge and to diversify its economy. Western property rights in innovation in the hands of crony capitalists can magnify the social harms of the patent system leading to high concentration of ownership of knowledge.

## Introduction

If Leo Tolstoy had written about innovation, his work might have started with “all innovation success stories are alike; each innovation failure fails in its own way”.^1^ Much scholarship has been written about the institutional failure of patents in developing countries.^2^ Almost nothing has been done about Russia.^3^ However, what is distinctive about the Russian innovation case is that it does not fit the category of a developing country. Russian innovation fails in its own way. On the one hand, Russia is a sophisticated technological power that demonstrated that it is capable of producing innovation. Russian human capital in mathematics and information technologies is among the best in the world.^4^ Russia leads in world exports of nuclear power plants.^5^ Recently, Russia manifested its innovation capacity in the pharmaceutical industry despite it being primarily generic and critically dependent on imported medicines.^6^ The 2020 surprise that came with the development of the Sputnik vaccine echoed the 1957 surprise from the Sputnik satellite, reminding the world once again that Russian technological power should not be underestimated.

On the other hand, the Sputnik vaccine is an outlier. Its development is a response to the health crisis and not an outcome of the market-based institutions for innovation. Behind the development of the Sputnik vaccine is the Gamaleya Institute, which for 100 years has been at the forefront of solving fundamental problems in microbiology, immunology, and virology and organizing mass vaccinations.^7^ Similarly, KoviVac, another Russian vaccine against Covid-19, was developed by the Chumakov Centre which successfully introduced the polio vaccine nearly 80 years previously.^8^ Russia’s breakthrough in Covid-19 vaccines development is the legacy of the alternative regulation of knowledge production that existed in the Soviet Union. Both the Gamaleya Institute and the Chumakov Centre developed Covid-19 vaccines relying on an extensive knowledge base accumulated long before Russia introduced market-based patent rules. But what might modern Russian pharmaceutical innovation be like if it could only be managed by institutions that make the best use of the Russian innovation capacity? Is Russia capable of advancing its domestic generic industry to an innovative level that will produce new molecules steadily?

Throughout its history, Russia has often experimented with institutions for innovation.^9^ Some experiments have been successful; others have failed. The institutional experiments of the Soviet period turned its innovation system into one of the two most productive innovation systems in the world up until the late 1980s.^10^ The Soviet period was a peak moment of Russian innovation. The number of inventions in the USSR was on par with the number of inventions in the U.S.^11^ And the Soviet pace of industrialization and economic growth has had few equals in world history.^12^

After the break-up of the Soviet Union, Russia once again experimented: it transplanted the globalized institution of patents and by doing this introduced high barriers to entry in innovation markets.^13^ Since then, its innovation performance has been declining. The number of inventions in modern Russia has plummeted and currently lags behind the number of inventions in the U.S. approximately ten-fold.^14^ The Russian economy has narrowed itself down to resource export: it is not diversified and is hugely dependent on world prices for oil and gas.^15^ At the moment, Russia finds itself squeezed between the resource curse and its unrealized potential to innovate. In other words, Russian innovation has entered the moment of what venture capitalists describe as “the death valley curve”.^16^

Russia needs to emerge from the other side of “the death valley curve”, and this matters both for the Russian people and for the rest of the world. For the Russian people, overcoming the innovation decline would result in economic and ideological gains: not only would Russians get a more sustainable model of economic growth, one more consistent with goals of sustainability, but they would also regain the image and pride of a nation capable of producing world-class innovation. For the rest of the world, Russian innovation capacity is important because it is a potential source of a more diversified economy and a tool for lifting Russia out of crony capitalism.^17^ A Russia free from the resource curse and crony capitalists would become a more stable international partner.

As mentioned, the most recent big experiment that Russia has undertaken with institutions for innovation has been to transplant the globalized institution of patents. Patents create barriers to entry in innovation markets. Sometimes these barriers are justified because they incentivize competition, but sometimes they are not because they stifle it. The difference between these outcomes lies in the broader institutional context, such as the presence or absence of well-functioning markets. The article argues that the globalized institution of patents has been compromised in Russia because the barriers to entry that patents create are not justified in the absence of well-functioning markets. The article demonstrates that the danger of the institutional mismatch is the lost opportunities for Russia to grow knowledge and to diversify its economy. The crony capitalist state will magnify the social harms of the patent system. Western property rights in innovation in the hands of crony capitalists can lead to a paradoxical effect of more concentrated control of innovation by the network of cronies which is a threat both for national welfare and the rest of the world.

The rest of the article is structured as follows. Section 2 explains the institutions for innovation that Russia had in the Soviet period before it entered the moment of “the death valley curve”. The main practical idea behind the Soviet innovation system was to make knowledge flow. This idea was realized through the alternative to patents, the system of inventor’s certificates, and the safeguards against the lock on future innovation. Section 3 examines the introduction of a new institution for innovation, the globalized institution of patents, and evaluates the dangers it engenders in the Russian context. Patents have created high barriers to entry in innovation markets and overruled the previous principle of free knowledge flow. The institution of patents is examined in the context of Russian crony capitalism. Section 4 paints optimistic and pessimistic scenarios of how Russia could deal with its moment of crossing (or not) “the death valley curve”. Section 5 concludes.

## Soviet Institutions for Innovation and Free Knowledge Flow

The Soviet innovation system, as mentioned, was one of the two most productive innovation systems in the world. Despite being isolated from foreign knowledge flows, the Soviet economy was able to make big jumps forward, such as the rapid transformation of an agricultural economy into an industrial one and ensuring self-sufficiency in heavy industry production.^18^ How was the Soviet Union able to make such big jumps?

Soviet scholars agreed with their Western counterparts that technological innovation was important for economic and societal progress. They disagreed however on the point of how knowledge flow should be organized in the economy: Soviets argued that the economy should be organized in a way to allow the *free* flow of inventions.^19^ There was a clear understanding in the Soviet Union that it would not be able to catch up with the West unless it lifted legal restrictions on knowledge flow. To put this idea into practice, the Soviets introduced an alternative to the patent system, the system of inventor’s certificates, as well as safeguards against the lock on future innovation which included broad exceptions to patentability and rules of discoveries.

### Inventor’s Certificates

The key characteristic of the Soviet innovation system was managing two parallel legal institutions for inventions: inventor’s certificates and patents. Soviet inventors could choose which of these legal institutions to use. Most applied for inventor’s certificates.^20^ The criteria for the grant of inventor’s certificates and patents were similar: the standard of invention was common for both legal institutions and consisted of novelty, industrial application, and from 1959 also usefulness.^21^ What was distinctive about inventor’s certificates in comparison to patents was that the former incentivized inventors to transfer the exclusive right in an invention to the state. In return, inventors received guaranteed monetary rewards and social benefits such as priority rights for promotion, education, accommodation, etc.^22^ In contrast, by applying for a patent, an inventor preserved the exclusive right but was not entitled to the mentioned benefits. The Soviet regulation of patents resembled the market-based regulation, in which a patent does not confer a reward but rather allows its holder to exploit the right to obtain the reward.

#### Liability Rule Instead of Exclusivity Rule

The transfer of an exclusive right to the state through the inventor’s certificate activated the rule known as the “liability rule”. Patents, on the other hand, activated the “exclusivity rule”. The distinction between liability rule and exclusivity rule is to be found in the classic article by Calabresi and Melamed.^23^ The liability rule underlying the inventor’s certificates meant that an inventor could not exclude third parties from using the invention but had to be compensated for such use. In the Soviet system, compensation took the form of material rewards and social benefits. The size of the material reward depended on the economic usefulness of the invention which was defined according to the government’s methodology.^24^ The measure was extensively implemented: by 1970, more than 76 million inventors and efficiency experts were rewarded.^25^ In contrast, the exclusivity rule underlying patents allowed the patent holder to exclude anyone from the use of the invention. No reward was guaranteed because the patent holder had to put the invention into production in order to obtain a reward. In this case, the size of the reward depended on the capacity to exploit the patent.

The same Soviet inventor could not benefit from both legal institutions on different inventions. If the inventor held both the patent and inventor’s certificate, the material rewards and social benefits of the latter were withdrawn. Such strictness could be explained by the need to protect knowledge flow from attempts to play the system, for example, by compromising the openness of some parts of a technology by keeping other parts exclusive. And the system generally was framed in such a way as to make the liability rule the preferred choice of inventors. Enforcing the liability rule underlying inventor’s certificates ensured faster and easier knowledge flow to the public domain.

#### Active Diffusion of Inventions

Inventor’s certificates eliminated legal restrictions on the use of knowledge. From the moment an inventor’s certificate was issued, any Soviet enterprise could implement the invention in their production.^26^ Nevertheless, although most inventions were free from legal restrictions on their use, the Soviets thought that passive knowledge flow was not enough. Their idea was to have knowledge actively diffused in the economy. For this purpose, in addition to state economic planning, the Soviets planned innovation.

In broad terms, the innovation cycle in the Soviet Union can be described as follows. Inventors, depending on whether they were employed or not, disclosed their initial idea to the enterprise or the NGO of All-Union Society for Inventors and Rationalizators (Efficiency Experts). This was the first step. The role of the first step was to help inventors technically (for example, to run experiments and develop a prototype) and legally (to draft and submit an application for an inventor’s certificate). Within enterprises, such work was done by bureaus for rationalization and inventiveness. These bureaus had to be established in each enterprise employing more than 500 people.^27^ The bureaus accumulated knowledge and competence relevant to their industry by cooperating with similar bureaus in other enterprises and R&D institutes and by monitoring new inventions in the industry.^28^ Many Soviet enterprises had in-house scientific-research laboratories (in 1967, there were 33,000 laboratories), and large enterprises established R&D institutes.^29^ The role of bureaus and R&D institutes was crucial: as Kojevnikov points out, in the Soviet Union production was subordinated to research institutes and not vice versa.^30^

The second step was the issuing of an inventor’s certificate by the Committee on Inventiveness. Formally, the Committee on Inventiveness was a predecessor of the modern Russian Patent Office, but the Committee’s role was much broader. In addition to examining applications for inventor’s certificates, the Committee on Inventiveness evaluated the usefulness of inventions. Usefulness was determined based on cumulative feedback that the Committee gathered from any enterprise or R&D institute.^31^ The Committee on Inventiveness also coordinated the mentioned bureaus within enterprises.

The third step was to include a new invention in state planning. The Committee on Inventiveness sent the information about the issued inventor’s certificates to the State Committee on Science and Technology.^32^ Relying on this information and also on the scientific findings of R&D institutes, the State Committee on Science and Technology sent their recommendations to the State Planning Committee. Then, every three months, the State Planning Committee sent plans for implementing new useful inventions to industrial ministries. These ministries converted these plans into specific assignments for the subordinated enterprises.^33^ This is how Soviet enterprises both contributed to and benefited from the free flow of inventions.

What the Soviet system of inventor’s certificates created was the public domain of inventions. One can also see it as an example of networked open source cooperation, one that anticipated the development of open source innovation in the West. The Soviet public domain was, however, different from the Western system of publication of patents. The Soviet public domain grew faster because inventions became available for broad use immediately after the inventor’s certificate was issued. The Western public domain was then and continues to be rather an informational public domain: it signalled about new inventions but also about the legal restrictions which accompany them. The Soviet system of inventor’s certificates was an active public domain, whereas the Western system of patents has been a passive public domain. Soviet scholars considered the inventor’s certificates to be a better institutional solution than patents because the former allowed broader and faster diffusion of inventions in the economy.^34^ The rate of diffusion of Soviet inventions was indeed high: in 1960, about 64% of all inventions and efficiency proposals were implemented in Soviet production, and there was constant pressure to increase this rate.^35^

In summary, the Soviet system of inventor’s certificates incentivized inventors to transfer the exclusive right to the state. The underlying rule of inventor’s certificates was the liability rule. This rule prevented inventors from excluding third parties from the use of the invention but ensured that the inventor received compensation in the form of material rewards and social benefits.^36^ Inventions under the system of inventor’s certificates entered the public domain almost immediately.^37^ A complex infrastructure was set up to actively diffuse knowledge among all the Soviet enterprises.

One might ask: if the system of inventor’s certificates functioned so well, why were patents still an alternative legal institution for inventions in the Soviet Union? A plausible explanation could be that the Soviets wanted to preserve a legal institution that would facilitate the import of foreign technology. For foreign inventors, preserving the exclusive right was more familiar than navigating the deeply national inventor’s certificates system.

### Safeguards Against the Lock on Future Innovation

#### Broad Exceptions to Patentability

In addition to the system of inventor’s certificates, ensuring free knowledge flow required a set of safeguards that prevented the lock on future innovation. Among the safeguards, there were broad exceptions to patentability. These were many, even though the share of patents in comparison to inventor’s certificates was very low. Exceptions in the pharmaceutical and IT industries were probably the biggest contrast with the modern globalized regulation of inventions. For instance, between 1924 and 1991, no product patents on pharmaceutical inventions were issued.^38^ This exception cleared the path for other inventors to innovate around pharmaceutical invention and to figure out more efficient processes for producing the same substance. Not allowing patents on pharmaceutical products was a practice that was followed by many countries in the 20th century. In the IT industry, exceptions to patentability extended to algorithms and business methods.^39^

#### Rules of Discoveries

Another safeguard against the lock on future innovation was a standalone legal institution of discoveries.^40^ Discoveries were defined as “the establishment of the unknown before, objectively existing regularities, characteristics, and phenomena of the material world”.^41^ The purpose of the legal institution of discoveries was to distinguish scientific advancements from inventions. A certificate of discovery, similar to an inventor’s certificate, entitled its holder to material rewards and social benefits, but no one obtained the exclusive right, including the Soviet state itself. The regulation of discoveries targeted the problem of “dual-use inventions” which embodied findings of basic science and industrially useful innovation.

In pursue of exporting an open knowledge culture, the Soviet Union was the principal advocate in the WIPO for the conclusion of an international treaty on discoveries.^42^ The proposal faced some hesitation because the majority of the countries did not provide protection of discoveries. Eventually, the Geneva Treaty on the International Recording of Scientific Discoveries was adopted in 1978, although with an explicit reservation that the registration of discoveries will be voluntary without any legal effect (e.g. on conferring economic rights to authors of discoveries except the fact of recognition).^43^ Discoveries recorded under the Treaty could be used without any restriction. The Treaty, therefore, became the first multilateral agreement facilitating access to scientific information.

To conclude, the key practical idea of the Soviet innovation system was an emphasis on knowledge flow. Under the system of inventor’s certificates, inventions entered the public domain faster and were used simultaneously by more enterprises than under the patent system. Broad exceptions to patentability and the regulation of discoveries created safeguards against the lock on future innovation. The Soviet innovation system was deeply national and was subordinated to the logic of the state-planned economy. After the break-up of the Soviet Union, Russia started to transition to a market economy and joined the processes of liberalization of world trade. Part of the deal to join the world trade club required Russia to transplant the globalized institution of patents which stood the cornerstone of its innovation system on its head.

## Russian Institutions for Innovation and Entering “the Death Valley Curve”

The break-up of the Soviet Union was a shock to its innovation system. The established links between R&D institutes and enterprises were broken as a result of chaotic privatization. The logic of state planning of innovation was abandoned. The country’s innovation performance plummeted. The Soviet idea of free knowledge flow was substituted by the idea of having property rights in innovation. It has now been 30 years since Russia introduced patents as the only legal institution for inventions. This section focuses on this change and analyses the role of patents in the current Russian context, which includes malfunctioning markets and crony capitalism.

### The Process of Legal Transplantation

In this section, I address how and why Russia substituted the idea of free knowledge flow with the idea of property rights in innovation. The break-up of the Soviet Union happened at a time when the world was engaged in the process of regulatory globalization. Regulatory globalization was framed as a condition for the liberalization of international trade under the WTO framework. Before concluding the Agreement on Trade-Related Aspects of Intellectual Property,^44^ the U.S. reached bilateral agreements with many countries aiming to raise their standards of intellectual property protection before the formal multilateral negotiations at the WTO. This process is well documented in the literature.^45^ The bilateral agreements and the TRIPS Agreement became the source of legal transplantation of intellectual property rules.^46^

Russia was no exception to this process.^47^ In 1990, when the Soviet Union still existed, the bilateral agreement with the U.S. required the Soviet Union to issue both product and process patents in all areas of technology.^48^ The “patents without discrimination” rule would later become the cornerstone of the TRIPS Agreement.^49^ Agreeing to this rule for the Soviet Union meant that it had to discard the system of inventor’s certificates and narrow down the exceptions to patentability which had included product patents in pharmaceuticals, algorithms and business methods patents.

After the break-up of the Soviet Union, Russia maintained the course that the U.S.–USSR bilateral agreement set out. Russia, similar to other post-Soviet countries, started wholesale legal reforms introducing market institutions. The U.S., in order to accelerate these reforms, promised to grant the most-favoured-nation principle if countries implemented legal reforms fast.^50^ In 2006, the U.S. concluded a bilateral agreement with Russia requiring it to “ensure that any changes in its laws, regulations and other measures made prior to its accession to the WTO … do not result in a lesser degree of consistency than exists on this date with the provisions of the TRIPS Agreement”.^51^ As a result, Russia adopted the standards of intellectual property protection that the TRIPS Agreement required long before its formal accession to the WTO which happened only in 2012. One of the key standards was introducing the globalized institution of patents which creates strong property rights in innovation with narrow exceptions.

### The Globalized Institution of Patents

Although patents existed in the Soviet Union as an alternative legal institution for inventions, their share in the overall numbers of inventions was marginal. As a result of the legal transplantation mentioned above, patents became the only legal institution for inventions in Russia. Introducing patents in what used to be the active public domain of inventions had retrospective and prospective effects. The retrospective effect was that the Soviet system of inventor’s certificates was abolished, and it became possible to exchange the inventor’s certificates which were still in force for patents.^52^ When such exchange occurred, it led to erecting barriers to entry where previously there were none.^53^

The prospective effect was that all new inventions became subject to property rights which delayed entry of knowledge to the public domain. The duration of the exclusive right was prolonged from 15 to 20 years.^54^ A broader subject matter became patentable: “a technical solution in any field that refers to a *product* (in particular, device, substance, microorganism strains, cell culture of plants or animals) or a *process* (a process of affecting a material object using material means)”.^55^ Such an extended scope of patentable subject matter cancelled the Soviet exception of not allowing product patents in pharmaceuticals. From 2014, in addition to product and process patents, “use patents” started to be issued.^56^ The further extension of the scope of patentable subject matter creates more barriers to entry and favours, in particular, patent holders in the pharmaceutical industry. They can accumulate large patent portfolios consisting of product, process, and now also use patents, all of which can relate to the same molecule. Patent portfolios create much higher barriers to entry than a single patent.

Soviet rules which prevented the lock on future innovation were erased. For instance, besides product patents in pharmaceuticals, patents on algorithms and business methods also started to be issued. The new rule only excludes patents on business methods and software if the patent application concerns these subjects as such.^57^ The way out, as the rule itself suggests, is to draft patent applications where these subjects are embedded indirectly, for example, in the form of a feature of a device. The legal institution of discoveries was also eliminated. Without discoveries, the line between basic science and commercially useful innovation became blurred. “Dual-use” innovation was gated by barriers to entry too.

In sum, when Russia transplanted the globalized institution of patents, it introduced barriers to entry in innovation markets. The extended term and scope of exclusivity, together with the narrowed exceptions to patentability, strengthened these barriers to entry. Under the new patent system, knowledge does not flow to the public domain until the exclusivity expires (potentially up to 20 years plus the patent term extension for pharmaceutical products). And successive patents on minor variations of an invention may prolong this beyond the term of any single patent. The public domain of knowledge thus grows much more slowly than under the system of inventor’s certificates.

Patents create barriers to entry in innovation markets.^58^ This is because patents, in fact, are rights which give monopoly pricing power and ability to prevent the follow-on innovation. Patent systems put a price on access to knowledge. The discussion then comes around to what extent such barriers to entry are necessary for innovation. If barriers to entry are too high, they hamper competition because the cost of entry rises and can become prohibitive.^59^ To date, there is no econometric evidence that the institutions that the TRIPS Agreement created have increased innovation.^60^ Patents are only one of the institutions for innovation and cannot be analysed outside the context of other institutions. The effectiveness of patents depends, as Maskus has noted, on other market factors.^61^ For example, it is reasonable to assume that barriers to entry are justified only if they are placed in well-functioning markets. Well-functioning markets are those where there is strong and dynamic domestic competition. In Russia though, markets are anything but well-functioning as the rest of the article demonstrates. The economy is not diversified and is governed by networks of crony capitalists. In these institutional settings, the role of barriers to entry can become especially dangerous, a fact that has been ignored in the arguments for patents.

### The State of Russian Innovation

The main problem of the Russian innovation system, which is recognized both inside and outside of Russia, is the “mismatch of the innovation cycle”. In other words, the government funding of R&D is used inefficiently, universities produce results unrelated to industrial needs, and the industry itself demonstrates little interest in innovation. The Russian 2016 Strategy for Scientific and Technological Development signals the weak cooperation between research and business and the lack of coordination between national, regional, industry-specific and corporate levels of funding of R&D.^62^ The 2016 Global Innovation Index Report also underlines the Russian problem of “fragmentation and lack of contingency between the components of the innovation system”.^63^ Poor interaction of key actors of the national innovation system is what precludes Russia from creating high-tech value chains. Taken independently, the actors of Russian innovation system are not weak, but what prevents them from interacting efficiently with each other is the environment of poorly functioning markets.

Well-functioning markets indeed did not spring up overnight after the system of state innovation planning was abolished. Most of the Soviet innovation infrastructure ceased to exist. The Soviet value chains were broken as a collapse of one enterprise launched a “domino effect”, leading to the collapse of associated laboratories and research institutes.^64^ As a result of privatization, new owners, having obtained discretion to decide on an enterprise’s structure, decided to liquidate bureaus for the facilitation of inventiveness, laboratories, and research institutes which had lost their purpose in a system of mismatched social practices and new formal rules.^65^ This is what happened at the level of enterprise.

At the level of government, the key Soviet organizations responsible for state planning of innovation were abolished (these include the State Committee on Science and Technology, the State Planning Committee, and the industrial ministries). The role of the few organizations that remained was narrowed down. For example, the NGO of the All-Union Society for Inventors and Rationalizators (Efficiency Experts) lost most of its members (from 14 million in 1988 to 100,000 in 2019).^66^ Today, it is rather a sad artefact of the past and no longer plays a visible role in the innovation cycle. Another example of narrowed organizational functionality is the Russian Patent Office, which formally is the successor of the Soviet Committee of Inventiveness. This Soviet body, as mentioned, used to coordinate the network for facilitation of inventiveness, to examine the usefulness of inventions, and to submit inputs for state planning of diffusion of inventions in the economy. In contrast, the functions of the Russian Patent Office are much narrower. It examines patent applications and issues patents, but it no longer plays a role similar to that of its predecessor in the innovation cycle.

The elimination of most Soviet organizations responsible for state innovation planning and narrowing down of their successors’ functionality were seen as necessary measures to create space for market-based innovation. However, the expectation that business would quickly take the state’s place in innovation markets was an illusion. Russian innovation could not operate in the vacuum fuelled only by the ideology that the market would sort out all the problems. Russian innovation needed real institutions which create the market dynamic. In an attempt to introduce such a dynamic, the Russian government started to borrow the Western models of organizations for development: technology parks, accelerators, and venture capital funds. These organizations for development are old wine in a new bottle. Funded and controlled by the state, they are the resurrection of the state-dominated innovation system, albeit with what is often seen as a poorer design than its Soviet predecessor.^67^

For example, *Skolkovo*, modelled on the Silicon Valley, was founded and fully funded by the Russian government. Its main function is to provide legal and business consulting services to the selected participants. The limited outreach of Skolkovo created “a gated community … almost closed-off to outsiders”.^68^ In regard to the venture capital market in Russia, as noted by Trofimova, its large share is associated with the state capital.^69^ One example is the *Russian Venture Company* which is 100% state-owned.^70^ Another example is the *Internet Initiatives Development Fund*, the capital of which is, presumably, a donation of a large oil and gas corporation.^71^ The donation followed soon after President’s Putin call for “looking together for the sources to fill the fund”.^72^ Today the Internet Initiatives Development Fund has grown to be one of the biggest players in the Russian venture capital market: it accounts for nearly half of venture transactions in Russia.^73^

Despite the similar appearance of Russian and Western organizations for development, the similarities are only superficial. What Russia does in practice through these organizations is to distribute state grants. In December 2020, the Russian government adopted an Act with a telling title: “On some issues of implementing *state support* of innovation activity, including by *venture and (or) direct financing* of innovative projects …” (emphasis added).^74^ The act introduces rules to evaluate the efficiency of state funding of innovation. One of the assessment indicators is the number of intellectual property rights, including patents, obtained as a result of the innovation projects funded by the state.^75^ However, the act shows no interest in who obtains these intellectual property rights. No requirements such as the local use of the invention or reasonable licensing terms are imposed on a patentholder who benefited from state funding. The efficiency of state funding is thus measured by the number of created barriers to entry in innovation markets. No attention is paid to who is behind those barriers and whether they are justified.

The patent system, as observed by Mazzucato, makes it possible to socialize costs and at the same time to privatize profits from innovation.^76^ Russia is no exception. The Russian grant system through the organizations for development has limited outreach and does not contribute much to the market dynamic. What it does instead is socializing costs of innovation while imposing no restrictions on extracting private profits. But the Russian problem is bigger than that. What happens if barriers to entry in innovation markets end up in the hands of crony capitalists?

### Russia in the Hands of Cronies

“Crony capitalism” defines situations when business elites operate relying on close personal ties with political elites.^77^ Cronyism usually implies corruption and rent-seeking associated with such businesses as oil and gas.^78^ Modern Russia is often characterized as a crony capitalist state.^79^ In 2016, Russia was ranked first in the *Economist*’s index of crony capitalism.^80^ The index captures the share of rent-seeking businesses in a country’s wealth. Forbes publishes the annual list of the world’s wealthiest public companies called “The Forbes Global 2000”.^81^ Russia’s top five companies in this list are from the oil and gas industry.^82^ There is a well-established literature documenting the Russian resource curse and how it affects the country’s capacity to develop market institutions.^83^

Patents were discussed earlier as barriers to entry in innovation markets. Crony capitalism is an institutionalized form of barrier to entry because new market entrants are normally excluded from the established crony networks by all means. Unlike patents, there is no term of expiry on a crony network. If exceptions are ever made, a new entrant is expected to reinforce the cronies’ network.

What is the role of property rights in crony capitalism? It seems natural to think that crony capitalism devastates the demand for property rights protection and other market institutions. However, the evidence does not support this assumption because there is a wide disparity in the levels of property rights protection in crony capitalist countries.^84^ In some countries, the high export prices on oil and gas even result in strengthening and consolidating property rights.^85^ In fact, cronyism in developed countries is part of the formal economy while in developing countries it is part of the shadow economy.^86^ Cronies in developed countries, and Russia is no exception, seek to legitimize their status by consolidating property rights. As Pistor argued, it is legal coding that generates wealth in assets.^87^ To apply her argument to Russian settings, it is legal coding that can legitimize the wealth of cronies. Patents are one of the tools which can protect cronies’ major concentrations of power.

The International Property Rights Index gives Russian patent protection a grade of 7.6, on the scale from the lowest (0) to the highest (10) levels, which globally positions Russia as the 45th country out of 129.^88^ Going back to Mazzucato’s argument, patents are often used to socialize costs and privatize profits which leads to value extraction. Value extraction is the ultimate goal of crony capitalists. The rational actor model would imply crony capitalists using patents not only to generate even bigger profits but also to slow down the development of innovation that threatens the stability of their business models. Diversifying the economy is a threat to crony capitalists, and this is where patents as an institution of public welfare can be dangerously compromised in Russia.

By now it should be clear that the case of Russian innovation is particular due to the rampant introduction of entry barriers in innovation markets and the emergence of crony capitalists who can make use of these barriers for their own benefit. Such a combination entails two big dangers. The first danger is lost opportunities for Russia to grow knowledge. In the absence of well-functioning markets, property rights in innovation have created unjustified barriers to entry in innovation markets. Under the new patent system, the knowledge does not flow as fast as it did in the Soviet system of inventor’s certificates. The stifled knowledge flow prevents the Russian economy, which is critically dependent on natural resources export, from diversifying. Russia cannot exercise the full potential of its highly qualified human capital and make use of its innovation capacity. The second danger is that strong property rights in innovation can be used to preserve the power of crony capitalist elites. This, again, not only hampers the Russian capacity to diversify the economy but also poses a military danger to the rest of the world. It is one thing when cronies extract rents from oil and gas, but it is entirely another thing when they use those rents to develop dual-use technologies such as artificial intelligence.^89^

Among the countries on top of the crony capitalist index, only Russia has the legacy of one of the two most productive national innovation systems in the world. This legacy combined with the unjustified barriers to entry in innovation markets can lead to the high concentration of ownership of innovation. The globalized patent rules that Russia transplanted into its law are likely to be used to reinforce the power of a dangerous form of crony capitalism in which political elites extract rents through an innovation agenda. As a result, the high concentration of ownership of knowledge will hinder the development of open and effective competition that could otherwise resist the proliferation of cronyism. In a crony capitalist state, social harms of the patent system are prone to amplify.

## Scenarios for the Future

Finding itself in a “death valley curve” moment, Russian innovation faces two scenarios, one optimistic and the other pessimistic. The positive scenario is that Russia overcomes the curve with regained pride, a diversified economy, and a promise of international stability. To do so, Russia has to adjust its patent system to grow and diffuse knowledge. Russia cannot go back to the Soviet system of inventor’s certificates but what it can do is to minimize the introduced barriers to entry in innovation markets. Minimizing barriers to entry would require, among other things, the broadening of exceptions to patentability, strengthening of the standard of invention, and preventing unjustified prolongation of the exclusive right. The goal of readjusting the Russian patent system should be to allow knowledge to grow and to flow. Those barriers to entry which Russia would decide to preserve should be re-evaluated against the other market institutions.

It has not been uncommon in the past for countries to readjust their patent systems multiple times following their progress of technological catch-up. Japan, for example, between 1950 and 1980 placed a great emphasis on knowledge flow and domestic competition. Its laws required Japanese firms which imported foreign technologies to share them with other domestic firms.^90^ It was only after this policy helped Japan to increase its innovation capabilities, that it was ready to strengthen its patent system to face a patent race with other technological powers.^91^ Although under the TRIPS Agreement the adjustments of a similar scope are no longer possible, countries still have some regulatory sovereignty in managing their patent systems.^92^

The pessimistic scenario is that Russia remains stuck in “the death valley curve”. Strengthening or simply preserving the existing barriers to entry in innovation markets in Russian settings would lead to conserving the crony elites who would use patent rights to legitimize their wealth. Patents in the hands of cronies magnify the tragedy of private value extraction at the cost of the public. For the Russian people, preserving the current system means the lost opportunity to grow knowledge and diversify the economy. The longer Russia stays in “the death valley curve”, the more it loses. While knowledge grows exponentially elsewhere, Russian barriers to entry in innovation markets stifle its capacity to innovate. The innovation gap between Russia and the developed countries widens, “the death valley curve” deepens, and it becomes harder to get out. These are the consequences for Russia. For the rest of the world, they are bitter too. At the first glance, one could see little value in saving the dying “Russian Bear” which for many was an irritation and threat during the Cold War. However, the danger is that barriers to entry in the hands of cronies have the potential to make the “Russian Bear” even more brutal. The Western system of property rights could lead to an unexpected effect of more concentrated control over innovation by the networks of Russian cronies, helping to reinforce their extractive business models and, consequently, conserve their power.

## Conclusion

The article has demonstrated why and how the Russian innovation system entered the moment of “the death valley curve”. Its predecessor, the Soviet innovation system, had been on another side of the valley, and its innovation performance was on par with other developed countries. The main idea of the Soviet innovation system was to make knowledge flow by relying on the system of inventor’s certificates and safeguards against the lock on future innovation. After modern Russia introduced patents as the only institution for inventions, it created high barriers to entry in innovation markets. These barriers to entry are not justified in the absence of other institutions like well-functioning markets. The article argued, therefore, that the globalized institution of patents has been compromised in Russia.

The danger of the institutional mismatch is the lost opportunities for Russia to grow knowledge and to diversify its economy. Another danger is that in the context of Russian crony capitalism, Western property rights in innovation can end up in the hands of cronies who use them to legitimize their wealth. These institutions thus can conserve crony elites but do not serve the Russian people. What the Russian innovation system does now is providing the opportunity to extract rents for multinationals and local cronies. Apart from reinforcing the rent-seeking activities, Western property rights in the hands of crony capitalists can magnify the social harms of the patent system by slowing down Russia’s technological progress and repressing the development of a more diversified economy.

To have a better future, Russia has to lift itself out of crony capitalism. Russia has no choice: it cannot return to the Soviet system, nor can it stay in “the death valley curve”. To climb out of the valley, with regained pride and the image of a nation capable of producing world-class innovation, Russia has to ask itself how to make knowledge flow in the system. A way to do so is to minimize the unjustified barriers to entry in innovation markets. The case of Russian innovation is worth keeping an eye on because, depending on what happens, it can be either a source of major negative disruption or positive stability for the rest of the world.

## Footnotes

1 A fantasy rephrasing of the beginning of Leo Tolstoy’s 1877 novel “Anna Karenina” which starts with “All happy families are alike; each unhappy family is unhappy in its own way”.

2 *See*, for example, Maskus and Reichman (2004), pp. 279, 302–303; Schneider (2005); For a contribution specific to pharmaceutical industry *see* Gamba (2017).

3 One of few works in the field is Payosova (2013).

4 Biagioli and Lépinay (2019), pp. 13–14.

5 Graham (2013), p. 96.

6 Rodwin et al. (2018), p. 203.

7 The Gamaleya National Center of Epidemiology and Microbiology: About us. https://sputnikvaccine.com/about-us/. Accessed 29 September 2021.

8 The Chumakov Federal Scientific Center for Research and Development of Immune-and-Biological Products of Russian Academy of Sciences. History (translation by author) http://www.chumakovs.ru/en/history/. Accessed 29 September 2021.

9 For a concise overview of the reforms *see* Ivanov and Voinikanis (2017).

10 Scerri and Lastres (2013), p. 12.

11 In 1987, the number of registered inventions in the Soviet Union was on a par with the U.S.: 83,700 and 82,900 respectively. Ivanov and Voinikanis (2017), p. 758.

12 Castells (1998), p. 10.

13 Lebedenko (2021), p. 314.

14 In 2019, Russia issued 34,008 patents, ten times less than the U.S. which issued 354,430 patents. Annual Report 2019 (translation by author). In: Russian Patent Office. https://rospatent.gov.ru/content/uploadfiles/otchet-2019-ru.pdf. Accessed 8 June 2021; U.S. Patent Statistics Chart Calendar Years 1963–2019. In: U.S. Patent and Trademark Office. https://www.uspto.gov/web/offices/ac/ido/oeip/taf/us_stat.htm. Accessed 8 June 2021.

15 For an overview of resource dependence problem, *see*, for example, Gel'man and Marganiya (2010).

16 “The death valley curve” is a metaphor used by venture capitalists to describe the period when start-ups begin to operate but do not generate any revenue yet. The metaphor seems to me very appropriate to describe the current state of Russian innovation when its innovation capabilities are not fully realized and do not result in a more diversified economy.

17 “Crony capitalism” defines instances when businesses operate relying on close relationships with politicians. Its manifestation in Russia is discussed below in Sect. 3.4.

18 Castells (1998), p. 26.

19 Yaichkov (1961), p. 4.

20 In 1976–1989, 90% of inventors applied for inventor’s certificates. The remaining 10% who applied for patents were mainly foreigners. Karpova (1992), pp. 26, 39.

21 Soviet Act on Inventions and Technical Improvements, Act No. 3/256 of 1931 (translation by author), paras. 45, 58; Soviet Act on Inventions and Technical Improvements, Act No. 448 of 1941 (translation by author), paras. 2, 29; Soviet Act on Discoveries, Inventions and Efficiency Proposals, Act No. 435 of 1959 (translation by author), para. 3; Soviet Act on Discoveries, Inventions, and Efficiency Proposals, Act No. 584 of 1973 (translation by author), para. 21.

22 One of early acts establishing the benefits is Soviet Act on Inventions and Technical Improvements, Act No. 3/256 of 1931 (translation by author), paras. 95–104.

23 According to the authors, rules could be conceptually divided in three categories. The first category is based on the principle of exclusivity and aims to exclude any infringement of the right. The second category is based on the principle of liability and aims at compensating damages for infringement of the right. The third category is based on the idea that some rights are inalienable. If one applies this categorization to regulation of inventions, the first two approaches, liability rule and exclusivity rule, are applicable. Calabresi and Melamed (1972), p. 1092.

24 The rules for defining the size of reward were established by the state. The reward was usually calculated as a percentage of economized resources which the invention helped to save during the first year of its use. Rewarding the inventor was a step in an algorithm that each enterprise followed while implementing an invention. There were a number of regulations, *see*, for example, Soviet Act on Reward for Inventions, Technical Improvements, and Efficiency Proposals, Act No. 1904 of 1942 (translation by author), para. 9.

25 Ivanov and Voinikanis (2017), p. 760.

26 The Soviet public domain was limited to the Soviet territory. The term of the exclusive right of the Soviet state was 15 years. Outside the Soviet territory, inventor’s certificates were licensed similarly to patents. In fact, from 1965 the Soviet Union was part of the 1883 Paris Convention for the Protection of Industrial Property, which implies that managing two parallel legal institutions for inventions did not prevent it from participating in international technology transfers.

27 Soviet Act on Inventions and Technical Improvements, Act No. 3/256 of 1931 (translation by author), para. 14.

28 *Ibid*., paras. 15–16.

29 In 1963, enterprises with fewer than 50 employees constituted 15% of all enterprises in the USSR. In the U.S. at the same time the respective number was 85% and in Japan 95%. Soviet enterprises with more than 500 employees constituted 24%, while in the U.S. 1.4% and in Japan 0.3%. Berliner (1978), pp. 33–34.

30 Kojevnikov (2004), p. 44.

31 Soviet Act on Discoveries, Inventions and Efficiency Proposals, Act No. 435 of 1959 (translation by author), para. 37.

32 The State Committee for S&T was operative from 1948 to 1991. It advised the government on the technological policy, coordinated the work of R&D institutes, distributed funds, established new scientific organizations. It selected major research projects developed by the Academy of Sciences of the USSR, union republic academies of sciences, and research organizations and handed them over to the industrial ministries to be worked out in their specialized departments. Berliner (1978), pp. 33–34.

33 Soviet Act on Discoveries, Inventions and Efficiency Proposals, Act No. 435 of 1959 (translation by author), para. 62.

34 Dozorcev (1969), p. 21.

35 Garmashev (1962), p. 23.

36 Soviet scholars considered such allocation of rights and obligation of inventors to be a good balance between public and individual interests. Yaichkov (1961), p. 14.

37 Balz (1975), p. 73.

38 Soviet Act on Patents on Inventions, Act of 1924 (translation by author), para. 1; Soviet Act on Inventions and Technical Improvements, Act No. 3/256 of 1931 (translation by author), para. 3; Soviet Act on Inventions and Technical Improvements, Act No. 448 of 1941 (translation by author), para. 2; Soviet Act on Discoveries, Inventions and Efficiency Proposals, Act No. 435 of 1959 (translation by author), para. 4; Soviet Act on Discoveries, Inventions, and Efficiency Proposals, Act No. 584 of 1973 (translation by author), para. 25.

39 Soviet Act on Discoveries, Inventions, and Efficiency Proposals, Act No. 584 of 1973 (translation by author), para. 21; Soviet Law on Inventions in the USSR, Law No. 2213-I of 1991 (translation by author), Art. 1, para. 3.

40 The Soviet institution of discoveries existed from 1959 to 1991.

41 Soviet Act on Discoveries, Inventions and Efficiency Proposals, Act No. 435 of 1959 (translation by author), para. 2.

42 WIPO (1981) Records of the Diplomatic Conference for the Conclusion of a Treaty on the International Recording of Scientific Discoveries, 1978. Geneva p. 80. https://www.wipo.int/edocs/pubdocs/en/wipo_pub_338.pdf. Accessed 29 September 2021.

43 *Ibid*.

44 Agreement on Trade-Related Aspects of Intellectual Property Rights, Marrakesh Agreement Establishing the World Trade Organization, done at Marrakesh, 15 April 1994, Annex 1C, 33 I.L.M. 1197 (hereinafter TRIPS Agreement).

45 Drahos (2004), pp. 407–409.

46 Papadopoulou (2016), p. 897.

47 Payosova (2013), p. 232.

48 Union of Soviet Socialist Republics – United States Agreement on Trade Relations, done at Washington, 1 June 1990; 29 I.L.M. 946. Art. VIII, para. 2(d).

49 TRIPS Agreement, Art. 27.1.

50 Pitta (1992), p. 501.

51 United States – Russia Bilateral Market Access Agreement, Side Letter on Intellectual Property Rights, done at Washington, 19 November 2006, https://ustr.gov/archive/assets/World_Regions/Europe_Middle_East/Russia_the_NIS/asset_upload_file148_10011.pdf. Accessed 8 June 2021.

52 Russian Act on Rules of Exchanging the Abolished Inventor’s Certificates for Patents, Act No. 35 of 1993 (translation by author), para. 1.

53 The effects and implications of the transformation of inventor’s certificates into patents have not been systematically assessed in the literature. Some authors argued that only one in a hundred of inventor’s certificates were exchanged to patents, possibly due to economic stagnation and lack of opportunities for commercialization in the early years of the transition to a market economy. *See*, for example, Dezhina and Leonov (2003), pp. 15–16.

54 Soviet Law on Inventions in the USSR, Law No. 2213-I of 1991 (translation by author), Art. 3, para. 3.

55 Russian Patent Law, Law No. 3517-1 of 1992 (translation by author), Art. 4, para. 1.

56 “Use patents” refer to the use of a product or a process according to the specific function, i.e. the use of a pharmaceutical substance for a particular disease treatment. Russian Civil Code, Part IV, Law No. 230-FZ of 2006 (translation by author), Art. 1350, para. 1.

57 Russian Patent Law, Law No. 3517-1 of 1992 (translation by author), Art. 4, para. 2; Russian Civil Code, Part IV, Law No. 230-FZ of 2006 (translation by author), Art. 1350, paras. 5 (4, 5).

58 For a brief overview of the debate *see* Heger and Zaby (2018), p. 175.

59 Caenegem (2007), p. 19.

60 Maskus (2017), p. 8.

61 *Ibid*., p. 8.

62 Russian Act on Strategy for Scientific and Technological Development, Act No. 642 of 2016 (translation by author), para. 11.

63 Gokhberg and Roud (2016), p. 159.

64 Kamko and Kirdina-Chandler (2018), pp. 152–153.

65 *Ibid*., p. 153.

66 Structure of the All-Russian Society of Inventors and Rationalizators (Efficiency Experts) (translation by author). In: All-Russian Society of Inventors and Rationalizators (Efficiency Experts). http://www.ros-voir.ru/ru/structure-page. Accessed 8 June 2021.

67 As Kuznetsova argues, “A specific feature of the Russian situation is the fact that the government’s influence over R&D and the NSI was predominant under both the ‘totalitarian’ Soviet regime; and in democratic Russia. … The result of the 20-year period of reform is paradoxical – a lot of good things inherited from the Soviet era were abandoned and rejected while the ‘Soviet’ R&D model’s features that the reformers were set to transform still largely remain in place”. Kuznetsova (2013), p. 88.

68 The size of the Russian venture market remains meagre: 172 million USD were invested in Russian enterprises in 2018. Simonova (2019), p. 189.

69 Trofimova (2017), p. 398.

70 Kamko and Kirdina-Chandler (2018), p. 156.

71 Suharevskaja 2016 Oil and Gaz Venture: How the Internet Initiatives Development Fund Works (translation by author). RBC. https://www.rbc.ru/magazine/2016/05/570fa16e9a794781cb616fa0. Accessed 8 June 2021.

72 2012 Putin Instructed to Issue Hundreds of Millions for the Internet for the “Creative Class” (translation by author). CNews.ru. https://www.cnews.ru/news/top/putin_rasporyadilsya_vydat_sotni_millionov. Accessed 8 June 2021.

73 The share of the Fund’s investment from 2014 to 2018 in the overall Russian venture investment was between 40–45%. Annual Report on the Activity of the Internet Initiatives Development Fund in 2018 (translation by author). In: Internet Initiatives Development Fund 2018, p. 7.

74 Russian Act on State Support of Innovation, Including Venture and (or) Direct Funding of Innovation Projects, Act No. 2204 of 2020 (translation by author).

75 *Ibid*., para. 28.

76 Mazzucato (2018), pp. 191, 202.

77 Prabhakar (2017), p. 91.

78 Aligică and Tarko (2015), p. 32.

79 For a recent contribution *see* Åslund (2019).

80 2016 Our Crony-Capitalism Index: The Party Winds Down. *The Economist*. https://www.economist.com/international/2016/05/07/the-party-winds-down. Accessed 8 June 2021.

81 2020 Global 2000: The World’s Largest Public Companies. *Forbes*. https://www.forbes.com/global2000/#2b4b6b05335d. Accessed 8 June 2021.

82 According to the list of 2020, *see ibid*.

83 *See* Gel'man and Marganiya (2010).

84 Zaostrovtsev (2010), p. 83.

85 *Ibid*., p. 73.

86 Aligică and Tarko (2015), p. 39.

87 Pistor (2019), p. 19.

88 2020 International Property Rights Index: Russia. In: Property Rights Alliance. https://www.internationalpropertyrightsindex.org/country/russia. Accessed 8 June 2021.

89 The development of Russian AI is led by the state-owned bank, the head of which is close to the political elites. Petrella et al. (2021), pp. 82, 100.

90 Odagiri et al. (2010), pp. 112–113.

91 *Ibid*., pp. 118–119.

92 This policy space has been clarified in Hilty et al. (2014); *see also* Reichman (2014), p. 142.

## References

[CR1] Aligică PD, Tarko V (2015) Capitalist alternatives: models, taxonomies and scenarios. Routledge, Taylor & Francis Group, Abingdon, Oxon

[CR2] Åslund A (2019). Russia’s crony capitalism: the path from market economy to kleptocracy.

[CR3] Balz MW (1975). Invention and Innovation under Soviet Law: a comparative analysis.

[CR4] Berliner JS (1978). The innovation decision in Soviet industry.

[CR5] Biagioli M, Lépinay VA, Biagioli M, Lépinay VA (2019). Introduction: Russian economies of codes. From Russia with code: programming migrations in post-Soviet times.

[CR7] Calabresi G, Melamed AD (1972). Property rules, liability rules, and inalienability: one view of the cathedral. Harv Law Rev.

[CR8] Castells M (1998). End of millennium.

[CR9] Dezhina IG, Leonov IF (2003) Intellectual property in Russia: the problems of state regulation (translation by author). Innovacii 14–20

[CR10] Dozorcev VA (1969). Legal status of inventor’s certificate under the new system of planning and economic incentivizing (translation by author).

[CR11] Drahos P (2004). Intellectual property and pharmaceutical markets: a nodal governance approach. Temple Law Review.

[CR12] Gamba S (2017). The effect of intellectual property rights on domestic innovation in the pharmaceutical sector. World Dev.

[CR13] Garmashev AF (1962) Inventiveness and its role in technical progress. In: Garmashev AF (ed) Inventiveness and efficiency in the USSR (translation by author). Profizdat, Moscow

[CR14] Gel'man V, Marganiya O (2010). Resource curse and post-Soviet Eurasia: oil, gas, and modernization.

[CR15] Gokhberg L, Roud V (2016) How to design a national innovation system in a time of global innovation networks: a Russian perspective. In: Dutta S, Lanvin B, Wunsch-Vincent S (eds) The global innovation index 2016: winning with global innovation. Cornell University, INSEAD, and WIPO, Ithaca, USA; Fontainebleau, France; and Geneva, Switzerland, pp 159–166

[CR16] Graham LR (2013). Lonely ideas: can Russia compete?.

[CR17] Heger D, Zaby AK (2018) Patent breadth as effective barrier to market entry. Economics of Innovation and New Technology. 27:174–188

[CR18] Hilty RM, Lamping M, Burk DL, et al. (2014) Declaration on patent protection: regulatory sovereignty under TRIPS. Max Planck Institute for Innovation and Competition, Munich

[CR19] Ivanov A, Voinikanis E (2017). A century of experimentation: institutional approaches to knowledge production in Russia from 1917 to 2017 and their implications for IP and competition equilibrium. The Antitrust Bulletin.

[CR20] Kamko EV, Kirdina-Chandler SG (2018). Institutional structure of Russian national innovation system: path-dependence effect (translation by author). Actual Problems of Economics and Law.

[CR21] Karpova NN (1992) Patent and licensing policy of the socialist states in 1976–1989 (translation by author). Nauchno-proizvodstvennoe ob’edinenie ‘Poisk’, Moscow

[CR22] Kojevnikov AB (2004). Stalin’s great science: the times and adventures of Soviet physicists.

[CR23] Kuznetsova T, Scerri M, Lastres HMM (2013). Russia. The role of the state: BRICS national systems of innovation.

[CR24] Lebedenko S, Ivanov A, Voinikanis E (2021). The effect of patent law on innovation: the problem of legal transplants. Patent Law alive and dead (translation by author).

[CR25] Maskus KE (2017) Cognitive dissonance in the economics of patent protection, trade and development. In: Ghidini G, Ullrich H, Drahos P (eds) Kritika: essays on intellectual property. Edward Elgar Publishing, Cheltenham, UK; Northampton, MA, USA, pp 1–21

[CR26] Maskus KE, Reichman JH (2004). The globalization of private knowledge goods and the privatization of global public goods. J Int Econ Law.

[CR27] Mazzucato M (2018). The value of everything: making and taking in the global economy.

[CR28] Odagiri H, Goto A, Sunami A (2010) IPR and the catch-up process in Japan. In: Odagiri H, Goto A, Sunami A, Nelson RR (eds) Intellectual property rights, development, and catch-up: an international comparative study. Oxford University Press, New York

[CR29] Papadopoulou F (2016) Legal transplants and modern lawmaking in the field of pharmaceutical patents – a way to achieve international harmonisation or the source of deeper divergences. IIC – International Review of Intellectual Property and Competition Law 47:891–911

[CR30] Payosova T (2013) Russian trip to the TRIPS: patent protection, innovation promotion and public health. In: Abbott FM, Correa CM, Drahos P (eds) Emerging markets and the world patent order. Edward Elgar Publishing, Cheltenham, UK; Northampton, MA, USA, pp 225–254

[CR31] Petrella S, Miller C, Cooper B (2021). Russia’s artificial intelligence strategy: the role of state-owned firms. Orbis.

[CR32] Pistor K (2019). The code of capital: how the law creates wealth and inequality.

[CR33] Pitta LA (1992). Intellectual property laws in the former Soviet republics: a time of transition. Santa Clara High Technology Law Journal.

[CR34] Prabhakar AC (2017) The current global recession: a theoretical and empirical investigation into developed and BRICS economies. Emerald, Bingley, UK

[CR35] Reichman JH (2014) Intellectual property in the twenty-first century: will the developing countries lead or follow? In: Cimoli M, Dosi G, Maskus KE, et al. (eds) Intellectual property rights: legal and economic challenges for development. Oxford University Press, Oxford, New York. doi:10.1093/acprof:oso/9780199660759.001.0001

[CR36] Rodwin VG, Fabre G, Ayoub RF (2018). BRIC health systems and big pharma: a challenge for health policy and management. Int J Health Policy Manag.

[CR37] Scerri M, Lastres HMM, Scerri M, Lastres HMM (2013). The state and the architecture of national systems of innovation. The role of the state: BRICS national systems of innovation.

[CR38] Schneider PH (2005). International trade, economic growth and intellectual property rights: a panel data study of developed and developing countries. J Dev Econ.

[CR39] Simonova A, Biagioli M, Lépinay VA (2019). Hackerspaces and technoparks in Moscow. From Russia with Code: programming migrations in post-Soviet times.

[CR40] Trofimova OE (2017). Investment of venture capital in Russia as an important element of transitioning to an innovative economy under sanctions. Stud Russ Econ Dev.

[CR6] van Caenegem W (2007). Intellectual property law and innovation.

[CR41] Yaichkov KK (1961). Invention and its legal protection in the USSR (translation by author).

[CR42] Zaostrovtsev A, Gel’man V, Marganiya O (2010). Oil boom: is it devastating to property rights and the rule of law?. Resource curse and post-Soviet Eurasia: oil, gas, and modernization.

